# A comparison of the temporal and spatial properties of trans-saccadic perceptual recalibration and saccadic adaptation

**DOI:** 10.1167/jov.20.4.2

**Published:** 2020-04-09

**Authors:** Matteo Valsecchi, Carlos Cassanello, Arvid Herwig, Martin Rolfs, Karl R. Gegenfurtner

**Affiliations:** Abteilung Allgemeine Psychologie, Justus-Liebig-Universität Gießen, Gießen, Germany; Department of Psychology, University of Bologna, Bologna, Italy; Department of Psychology, Humboldt-Universität zu Berlin, Berlin, Germany; Lise-Meitner-Gruppe Umweltneurowissenschaften, Max-Planck-Institut für Bildungsforschung, Berlin, Germany; Department of Psychology and Cluster of Excellence, “Cognitive Interaction Technology,” Bielefeld University, Bielefeld, Germany; Department of Psychology, Clinical Psychology, and Psychotherapy, University of Bremen, Bremen, Germany

**Keywords:** trans-saccadic size recalibration, saccadic adaptation, temporal dynamics, spatial specificity

## Abstract

Repeated exposure to a consistent trans-saccadic step in the position of the saccadic target reliably produces a change of saccadic gain, a well-established trans-saccadic motor learning phenomenon known as saccadic adaptation. Trans-saccadic changes can also produce perceptual effects. Specifically, a systematic increase or decrease in the size of the object that is being foveated changes the perceptually equivalent size between fovea and periphery. Previous studies have shown that this recalibration of perceived size can be established within a few dozen trials, persists overnight, and generalizes across hemifields. In the current study, we use a novel adjustment paradigm to characterize both temporally and spatially the learning process that subtends this form of recalibration, and directly compare its properties to those of saccadic adaptation. We observed that sinusoidal oscillations in the amplitude of the trans-saccadic change produce sinusoidal oscillations in the reported peripheral size, with a lag of under 10 trials. This is qualitatively similar to what has been observed in the case of saccadic adaptation. We also tested whether learning is generalized to the mirror location on the opposite hemifield for both size recalibration and saccade adaptation. Here the results were markedly different, showing almost complete generalization for recalibration and no generalization for saccadic adaptation. We conclude that perceptual and visuomotor consequences of trans-saccadic changes rely on learning mechanisms that are distinct but develop on similar time scales.

## Introduction

A wealth of objects populates our visual field, each creating a projection on the retina. Every time we move our eyes, the locations of these projections change. Most objects will now be seen at a different eccentricity relative to the situation preceding the eye movement. This poses at least two types of problems to the visual system. The first consists of being able to associate the pre- and postsaccadic locations, so that the representation of the visual scene does not have to be recomputed from scratch. Attention is likely to play a major role in how we solve this challenge. Predictive shifts of attention, tightly linked to saccadic programming, help maintain the continuity of the relevant objects in the scene (e.g., Rolfs, 2015; Rolfs, Jonikaitis, Deubel, & Cavanagh, 2011).

The second problem is one of accommodating the different representations that are associated with more or less peripheral viewing. Peripheral vision is characterized by less dense spatial sampling, resulting in reduced contrast sensitivity for higher spatial frequencies (Rovamo, Franssila, & Näsänen, 1992; Weymouth, 1958). At the same time, objects of equal retinal size are represented by a larger portion of visual cortex at smaller eccentricities (e.g., Daniel & Whitteridge, 1961). Additional differences emerge when it comes to color because spectral sensitivity changes in peripheral vision (Hansen, Pracejus, & Gegenfurtner, 2009; Weale, 1953). One mechanism that could reduce the perceived discrepancy between the peripheral and central input generated by the same object before and after a saccade is trans-saccadic integration (Ganmor, Landy, & Simoncelli, 2015; Herwig, 2015b; Wolf & Schütz, 2015). Other mechanisms, in turn, have a predictive nature. They involve either the extrapolation of foveal information to peripheral locations (Toscani, Gegenfurtner, & Valsecchi, 2017) or the integration of priors and templates in the peripheral percept (Galvin, O'Shea, Squire, & Govan, 1997; Galvin, O'Shea, Squire, & Hailstone, 1999; Valsecchi, Koenderink, van Doorn, & Gegenfurtner, 2018) so that the peripheral representation is more similar to the foveal one.

An additional mechanism that can reduce trans-saccadic discrepancy is the trans-saccadic recalibration of peripheral and central (i.e., foveal) appearance, that is, the fact that we can learn to map peripheral and central sensations by experience. A growing number of studies show that the appearance of peripheral stimuli is biased toward an expected central appearance when some arbitrary physical change is repeatedly and consistently associated with saccades. Features for which this is known to happen are spatial frequency (Herwig & Schneider, 2014; Herwig, Weiß, & Schneider, 2018), shape (Herwig, Weiß, & Schneider, 2015; Köller, Poth, & Herwig, 2018; Paeye, Collins, Cavanagh, & Herwig, 2018), and size (Bosco, Lappe, & Fattori, 2015; Valsecchi & Gegenfurtner, 2016). Similarly, chromatic hues can be associated to presaccadic locations in the visual field, which in turn biases color perception (Bompas & O'Regan, 2006a; Bompas & O'Regan 2006b), and even the same identity can be associated to different objects viewed centrally and peripherally (Cox, Meier, Oertelt, & DiCarlo, 2005).

The calibration of peripheral and central perceived appearance is a phenomenon that seems to exist at the boundary of two domains (Herwig, 2015a). On the one hand, it is a perceptual phenomenon, which can be both acquired and expressed without eye movements (Paeye et al., 2018; Valsecchi & Gegenfurtner, 2016). However, this form of calibration should take place most commonly due to trans-saccadic feedback. Indeed, the conditions under which recalibration occurred in the absence of saccades (i.e., when an object is seen first in the periphery, disappears and then reappears in the fovea, or moves toward the fovea, all during continued fixation) are unlikely to occur under natural viewing. When this form of learning takes place during saccades, the change in peripheral appearance can be considered akin to the perceptual changes that are associated with different forms of motor learning (Bosco et al., 2015; Ostry & Gribble, 2016). In particular, one finding by Bosco and colleagues (2015) is relevant in this sense. They used a trans-saccadic change that involved both a change in the size and a shift in the center of mass of the stimulus. This change produced both saccade adaptation and recalibration of perceived size, furthermore, the strength of saccadic adaptation and trans-saccadic recalibration correlated across observers, suggesting a possible functional connection between the two phenomena. Note, however, that even if perceptual and motor changes are produced within the same paradigm, and even if they do so in a correlated manner, they do not need to be manifestations of the same phenomenon, but simply rely on some shared processes, such as the detection of sensory change. Crucially, motor learning and perceptual changes can have a different time course, for example in locomotion adaptation (Leech, Day, Roemmich, & Bastian, 2018). Finding that the time course of trans-saccadic recalibration of perceived size markedly differs from the time course of comparable motor learning phenomena, such as saccadic adaptation, would support the conclusion that the two phenomena are independent and possibly different in nature.

Another domain in which perceptual and motor effects of trans-saccadic change might differ is spatial specificity. Although global adaptation can accrue from training multiple locations simultaneously (Rolfs, Knapen, & Cavanagh, 2010), adapting saccades aimed at one location induces an adaptation field (Alahyane, Devauchelle, Salemme, & Pélisson, 2008; Frens & Van Opstal, 1994; Hopp & Fuchs, 2004; Noto, Watanabe, & Fuchs, 1999) in which the amount of adaptation depends on the proximity of the saccade target with the adapted location. Thus the effects of saccadic adaptation are spatially localized. It is at the moment not clear whether and under what conditions this is true for trans-saccadic learning of perceptual features. On the one hand, Valsecchi and Gegenfurtner (2016) found that size recalibration transferred to locations at the same eccentricity but in the opposite hemifield. However, Herwig and colleagues (2018) found no transfer of spatial frequency learning between different saccade orientations.

None of the paradigms used to study trans-saccadic recalibration up to this point were specifically devised to quantify its temporal dynamics. Most of the previous studies involved a learning phase in which observers consistently experienced a trans-saccadic change (e.g., the peripheral stimulus became more rounded or its spatial frequency decreased during the saccade that foveated it), followed by a test phase that quantified the perceptual effect of the acquired association. All these studies demonstrated that these associations can be established within a session of fewer than 300 trials. The study by Valsecchi and Gegenfurtner (2016) combined the perceptual comparison and the saccade execution in a single trial, allowing for a better temporal resolution in the evaluation of the speed at which trans-saccadic learning recalibrated apparent size. Their findings suggested that a few dozen trials that induced a trans-saccadic change are sufficient to generate appreciable changes in perceived size, comparable with fast perceptual learning (Karni & Sagi, 1993). The maximum temporal resolution, however, was still limited by the fact that they used a size comparison task. Each trial only yielded a larger versus smaller response, and a value of equivalent size between center and periphery could only be computed over a range of trials.

In the present study, we modified a version of the paradigm introduced by Valsecchi and Gegenfurtner (2016), using an adjustment instead of the comparison task. This yielded a continuous measure of perceived size for each trial, avoiding the aggregation of responses over multiple trials. Furthermore, instead of introducing the trans-saccadic change gradually and then keeping it steady throughout a testing session (cf., Valsecchi & Gegenfurtner, 2016), we adapted a paradigm that Cassanello, Ohl, and Rolfs (2016) introduced to investigate the temporal properties of saccade adaptation (also see Cassanello, Ostendorf, & Rolfs, 2019). In this paradigm, the trans-saccadic size change followed a time-varying, sinusoidal evolution as a function of trial number. In a first experiment, we observed that a sinusoidal evolution of trans-saccadic size change produced a sinusoidal evolution of adjusted size, with a time course closely resembling the one that Cassanello, Ohl and Rolfs (2016) obtained in saccade adaptation. This result indicates that peripheral size recalibration and saccadic adaptation have comparable temporal dynamics when driven by stimuli modulated in a similar way.

In a second experiment, we investigated the spatial specificity of trans-saccadic recalibration and saccadic adaptation using a similar paradigm in terms of locations trained and tested, and in terms of the amount of training administered. The results clearly confirmed that learning transfers to a mirror horizontal location in the opposite hemifield, as suggested by Valsecchi and Gegenfurtner (2016), yet no trace of transfer was found for saccadic adaptation. Overall the results suggest that despite the fact that both forms of trans-saccadic learning take place with similar temporal dynamics, the mechanisms giving rise to them must be qualitatively different.

## Experiment 1: Time

### Methods

#### Participants

Fourteen observers participated in Experiment 1 (Time Experiment). Ten were women, and the mean age was 25.9 years. Seven were in the increase-first group and 7 in the decrease-first group (see Experimental design section). All participants were naive as to the purpose of the study and provided written informed consent in agreement with the Declaration of Helsinki. They were rewarded with either 8€/hour or with course credits. The study protocol was approved by the local ethics committee at the University of Gießen (LEK FB6 2017-08).

#### Setup

Stimuli were presented on a 22-in. Eizo CG223W 10-bit LCD monitor (Eizo Corporation, Hakusan, Japan), at a viewing distance of 40 cm. Eye movements were recorded at 500 Hz with an Eyelink II system (SR Research, Mississauga, Canada). Stimuli were generated and the experiment was controlled using MATLAB (MathWorks, Natick, MA), the PsychToolbox (http://psychtoolbox.org/) (Kleiner et al., 2007) and the Eyelink Toolbox (http://psychtoolbox.org/) (Cornelissen, Peters, & Palmer, 2002).

#### Procedure

**Figure 1.**
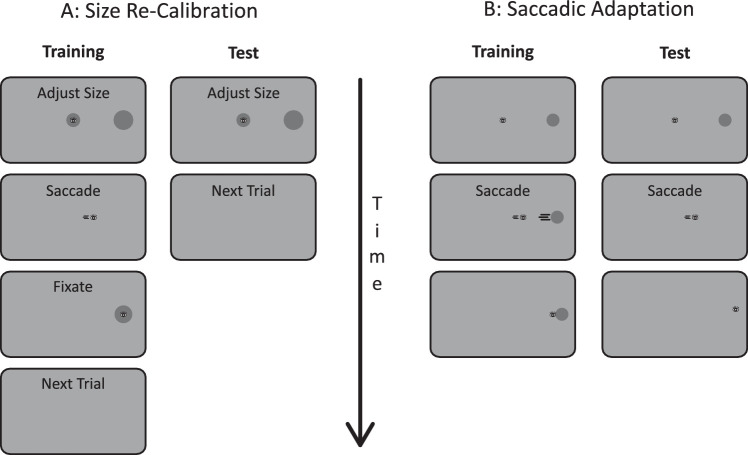
Trial procedures for all experiments. (A) Size recalibration trials. In Experiment 1 and in the training trials of Experiment 2, observers adjusted the central stimulus size to match the peripheral size. After confirming the adjustment, they were required to look at the peripheral stimulus and to keep fixating it for 1000 ms. Stimuli were only visible when gaze was located at the expected location (in the center before the saccade and on the formerly peripheral stimulus after the saccade). Notice that the peripheral stimulus always appeared on the same side of the screen for a given observer. The size of the peripheral stimulus presented after the saccade could be up to 15% smaller, identical, or up to 15% larger than the peripheral stimulus presented before. In the test trials of Experiment 2, both circles disappeared after the observer confirmed the size adjustment and the next trial followed. (B) Saccadic adaptation trials in Experiment 2. Observers had to saccade to the peripheral stimulus. In training trials, the stimulus remained on the screen (shifted to its postsaccadic location) after the saccade, in test trials it disappeared as soon as gaze left the fixation zone.

At the beginning of each trial, observers were required to fixate in the center of the screen, which prompted the appearance of two gray (8.79 cd/m^2^) discs over a lighter (25.55 cd/m^2^) gray background (Figure 1A). One circle was in the center of the screen, whereas the other one was located 20° left or right. The side was constant throughout the session and balanced between observers. The edge of the circles was smooth, following a cumulative Gaussian gradient with σ = 0.2°. While fixating the center of the screen, observers were required to adjust the size of the central stimulus to match the size of the peripheral one using the up and down arrow on the display computer keyboard. The speed of change accelerated as the observer kept a given button pressed (by 0.004 pixels every monitor refresh, starting from 0.001 pixels). This ensured that the observers could obtain subpixel changes in size with short button presses but also move relatively quickly if they depressed the button for longer times. In each trial, the initial radius of the central stimulus was picked from a flat distribution between 0.069° and 2.24°. Once they reached a satisfactory match, the observers had to press the enter key to confirm the adjusted size before making an eye movement toward the peripheral circle. Observers had unlimited time to perform the match, but after 30 seconds, a notice appeared prompting them to speed up their decision. At the first available monitor refresh after gaze left a 1.5° radius area around the fixation point, both circles disappeared. After 50 ms, assuming that the gaze was now within 3° from the center of the lateral circle, only the lateral circle appeared. Observers were also required to wait at least 1 second before performing the eye movement, so that the saccade event was temporally decoupled from the keypress. If observers tried to look before 1 second, both stimuli disappeared, and the observer had to look back to the central position. When they landed there, both the central and peripheral stimuli were presented again. The trial was concluded as observers fixated the lateral stimulus for 1000 ms. When the formerly peripheral stimulus reappeared in the fovea after the saccade, its size could be identical, up to 15% smaller in radius or up to 15% larger in different phases of an experimental session. Notice that the display was gaze-contingent and if observers blinked or failed to fixate within 1.5° from the center of the screen before the saccade, or within 3° of the lateral circle center after the saccade, the circles disappeared, and a small red dot indicated the required fixation location.

#### Experimental design

Each observer underwent a session of 300 trials, which lasted from 75 to 120 minutes depending on the observer´s speed. Recording was terminated and data were discarded for one participant who was unable to terminate the experiment within 120 minutes. Two parameters varied between trials: the magnitude of the trans-saccadic change that was applied to the lateral stimulus, and the size it had when presented in the periphery before the saccade. For all observers the session started with 50 trials in which no trans-saccadic change took place, that is, the stimulus reappeared after the saccade with the same radius it had before. In the next 200 trials, we introduced trans-saccadic change, going through two full sinusoidal cycles with peaks at 15% increase and 15% decrease (Figure 3, dashed lines). For seven observers the first peak was positive (increase-first group), for the remaining seven the first peak was negative (decrease-first group). The presaccadic peripheral radius had always one of five possible values (1.319°, 1.396°, 1.474°, 1.552°, or 1.629°) changing pseudorandomly between trials so that all sizes were presented twice every 10 trials.

### Results

#### Adjustment accuracy

**Figure 2.**
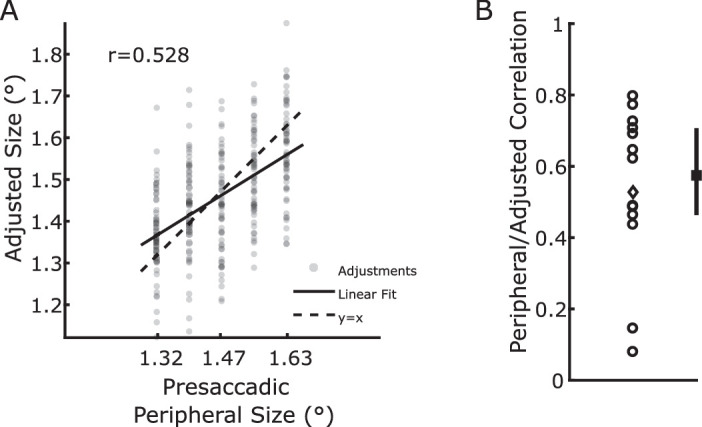
(A) Example of correlation between presaccadic peripheral size and adjusted size in one example observer. Each point represents one trial. (B) Correlation coefficients for all observers. Each circle represents one observer, the diamond represents the participant whose data are plotted in panel (A). The solid line represents the median, first quartile, and third quartile. Except for two observers, the correlation is relatively high, suggesting that most observers were able to perform the task. The data from the two observers who had correlations below 0.2 have been discarded before subsequent analyses.

The first analyses of the data aimed at evaluating to what extent the observers were able to perform the task of adjusting the central stimulus in accordance with the peripheral stimulus. The results are depicted in Figure 2. In the case of most observers the peripheral and adjusted size values correlate with coefficients larger than 0.4, indicating that observers successfully relied on the peripheral stimulus size for their adjustments. Two observers had very low correlation values, below 0.2, indicating that they were very poor at either processing the size of one or both of the stimuli, or at adjusting the central size. The data of these two observers appear as clear outliers in Figure 2B, and were removed from further analyses.

#### Temporal evolution of adjustments

**Figure 3.**
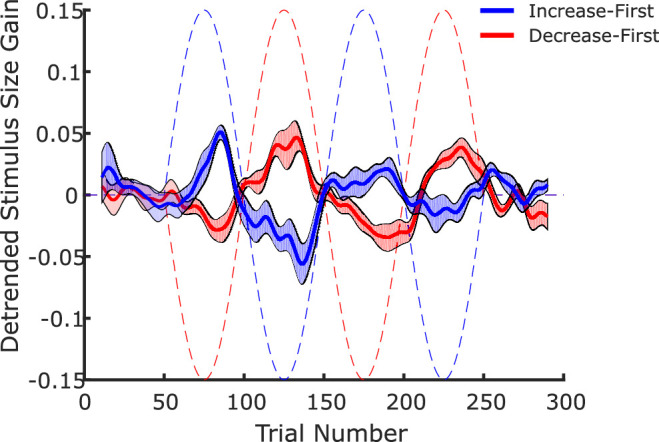
Time course of the trans-saccadic size change (dashed lines) and of the average adjusted size gain (solid lines) for the two groups of observers. The shaded areas represent the average value ± *SEM* across observers. The time series of each observer's adjustments has first been linearly detrended. Subsequently the overall time series of each observer has been smoothed using a Gaussian kernel with *SD* = 4 trials. Note that the counterphase oscillation of the physical change is mirrored in the counterphase oscillation of the adjusted size.

Before proceeding with the analysis of the temporal evolution of peripheral size recalibration, we computed the gain of the calibration as the percent ratio of the adjusted size of the central stimulus relative to the peripheral presaccadic size in each trial. The gain values were then linearly detrended for each observer, so as to get rid of any possible general tendency to decrease or increase in the adjusted values as the session progressed. The slope of this linear trend was on average positive (mean 0.010 and *SD* 0.015 gain units/100 trials) in the decrease-first group, and negative (mean –0.015 and *SD* 0.023 gain units/100 trials) in the increase-first group. This difference is statistically significant (*t*(10) = 2.28, *p* = 0.046), and can be explained by the fact that a trend with an absolute slope of 0.021 gain units/100 trials is present in the timeline of the physical stimulus change as well (positive in the decrease-first timeline and negative in the increase-first) because of the low number of cycles. This indicates that part of the recalibration effect might be discounted in our paradigm by the linear detrending, and the effects that we report in the following analyses are a conservative estimate of the overall impact of trans-saccadic change. Figure 3 depicts the overall time course of the adjustments after linear detrending for both groups. Clearly, the sinusoidal oscillation of the trans-saccadic size change had an impact on the adjustment gain, which also oscillated, albeit with a smaller amplitude and with a lag of a few trials.

To estimate the lag and amplitude of the induced oscillation for each observer, we fitted a simple descriptive model to the detrended gain values between trials 50 and 250:

$$\begin{array}{c} Gain_{n}=\left\{\begin{array}{c} Amplitude\;\cdot \;\sin\left(\frac{2\pi }{100}\left(n-50-Lag\right)\right),\;\;\;n>50+Lag\\ 0,\;\;n\leq 50+Lag \end{array}\right. \end{array}$$

Notice that a hypothetical amplitude of 1 and lag of 0 would predict the exact time course of trans-saccadic size change in the increase-first group, whereas an amplitude value of –1 and a lag of 0 would reproduce the time course of the decrease-first group.

**Figure 4.**
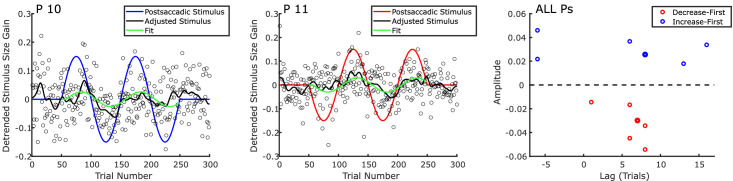
Left and center panels: examples of fitting procedure for individual observers (#10 and #11). The blue and red lines indicate the time course of the trans-saccadic change (blue and red for increase-first and decrease-first, respectively). Each dot indicates the adjustment in a single trial, the black line represents the smoothed (Gaussian kernel with *SD* = 4 trials) time course of size adjustments. The green line is the model fit. Right panel: amplitude and lag parameters for each observer in the decrease-first and increase-first groups (right). The thicker markers are for observers (#10 and #11). Note that in every case the sign of the amplitude corresponds to the one expected based on the time course of the physical manipulation in the respective group.

The model was fit using the least squares criterion and the *fminsearch* algorithm in MATLAB. Values of lag were constrained to be between –25 and 25 trials. All integer values between –25 and 25 were used as seeds for each observer, and the best fit of all was chosen. The amplitude values were not constrained in the fitting procedure. Note that a 50-trials shift is equivalent to a change in sign of the amplitude, so values of lag extending over a 50-trial window would be redundant. We decided to center the window on 0, despite the fact that a trans-saccadic change is unlikely to influence the adjustment of a preceding trial, as we wanted to avoid biasing our fitting procedure against very short lags. Examples of the data and of the corresponding individual fit functions are shown in Figure 4 (left and center panels).

The model fit parameters are depicted in the right panel of Figure 4. Note that, as expected, all time series from the increase-first group were fit with positive amplitude values, whereas all amplitude values in the decrease-first group were fit with negative amplitude values. The amplitude values were significantly different between groups (*t*(10) = 9.570, *p*<.001). The average absolute amplitude value was 0.0313, which amounts to 20.9% of the maximum expected amplitude that would have been 0.15. The lag values are on average 5.58 trials, although there was considerable variability between observers. Note that two observers were fitted with negative lag amplitudes. This is probably the result of noisy data, as it seems unlikely that observers were able to anticipate the sinusoidal trajectory of the trans-saccadic change that they were exposed to for only two full cycles.

## Experiment 2: Space

### Methods

#### Participants

Twelve observers participated in Experiment 2 (Space Experiment). Ten were women, and the mean age was 23.8 years. All participants were naive as to the purpose of the study and provided written informed consent in agreement with the Declaration of Helsinki. They were rewarded with either 8€/hour or with course credits. The study protocol was approved by the local ethics committee at the University of Gießen (LEK FB6 2017-08).

#### Setup

The setup of the study was identical to that in Experiment 1.

#### Procedure

Experiment 2 involved separate trans-saccadic recalibration and saccadic adaptation sessions. In each session there were separate training and test trials (see Experimental design section).

The procedure in the training trans-saccadic recalibration trials was identical to the one of Experiment 1 (Figure 1A), with the difference that the trans-saccadic size change could only take values of 0, +15%, and –15%. For each observer the target in training trials was always located on the same side of the screen. Test trials differed in three aspects: (a) the peripheral circle could be located randomly at the trained location or at the mirror location on the other side of the screen; (b) both circles disappeared immediately after the enter key was pressed to confirm the size adjustment; (c) no saccade was performed.

Saccadic adaptation trials (Figure 1B) were initiated by the observers by pressing the spacebar, which prompted the appearance of a small (0.16° radius) black (0.34 cd/m^2^) fixation dot in the center of the screen. After 1 second, a gray circle (1.474° radius) appeared centered 16° right or left of the fixation point, and observers were required to make a saccade toward it within one second; otherwise a warning was issued, and the trial was discarded. The side of the saccadic target was always the same for one observer in training trials. As soon as gaze moved further than 1.5° away from the fixation point, the procedure diverged between training and test trials. In training trials the target circle remained on the screen for one second, either at the same location or displaced 2.4° closer or further away from the original fixation point. In test trials the stimulus disappeared. Test trials also differed from training trials in that the target was randomly located left or right of fixation.

#### Experimental design

**Figure 5.**
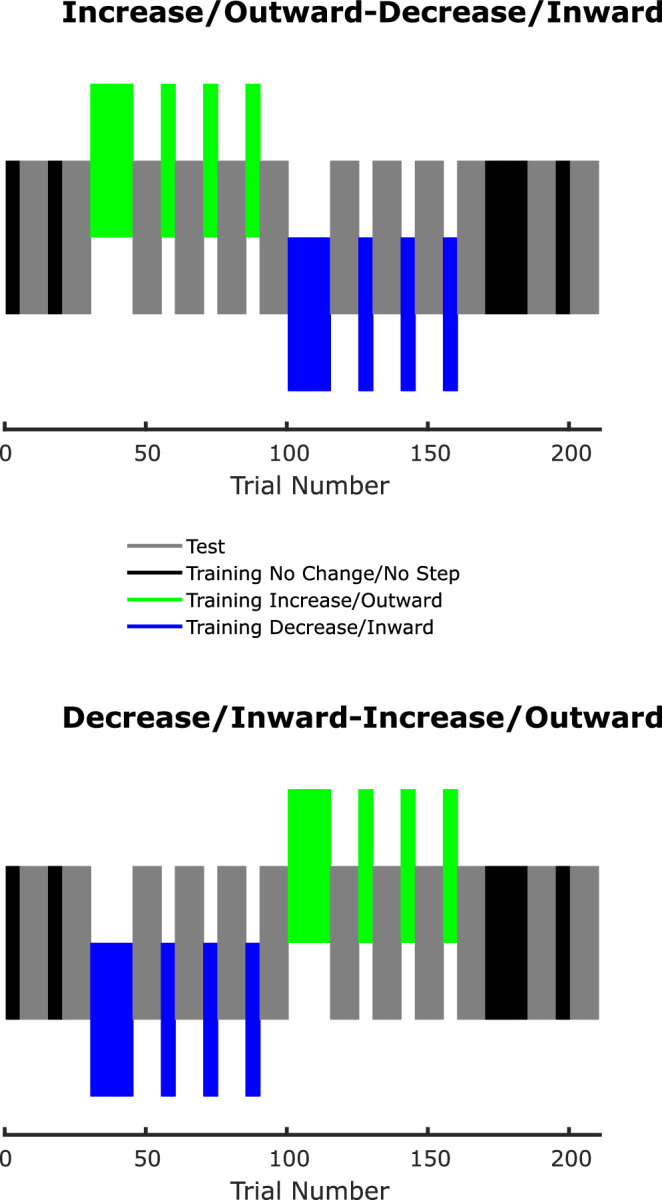
Experimental design. Blocks of the different types of trials are color coded. Notice that blocks of training and test trials were interleaved throughout the experiment. The training trials in which the stimulus size increased trans-saccadically could either precede or be performed after the ones in which the stimulus size decreased. Both sequences were performed by each observer on different days. The same scheme was used in the saccade adaptation trials, whereby no change would correspond to no step, increase to outward step, and decrease to inward step.

The experimental design was the same in both the trans-saccadic recalibration session and the saccadic adaptation session and is detailed in Figure 5. Each observer was tested twice, on separate days. Each test included a session of trans-saccadic recalibration followed by a saccadic adaptation session. Each session consisted of 210 trials: 120 test trials divided in 12 blocks of 10 trials and 90 training trials. Each block of test trials was preceded by a block of 5 or 15 training trials. In the training trials of the trans-saccadic recalibration session, the intrasaccadic change could either be absent, a 15% increase in diameter, or a 15% decrease in diameter. The first and last two blocks of training trials had no trans-saccadic change, the rest was divided equally between increase and decrease blocks, with the order being alternated between the two testing days, and the order of the initial day alternated independently for the trans-saccadic recalibration and saccade adaptation sessions between observers. In the case of the trans-saccadic recalibration, all of the five possible peripheral sizes were shown in random order every five training trials. Every block of 10 test trials had stimuli of all five sizes and on both sides randomly interleaved. In the case of saccadic adaptation, instead of no change, increase and decrease, the manipulation in training trials was no step, outward step, and inward step, respectively. The size of the saccadic target was again chosen randomly in every trial from the same set of five possible sizes.

#### Data analyses

The initial steps of data analyses for the trans-saccadic recalibration trials were the same as in Experiment 1. After having ensured that for each observer the adjusted sizes correlated with the target sizes over all trials (minimum value of *r* was 0.429), we proceeded to transform the values from raw adjustments into adjustment gain by computing the ratio between adjusted central size and peripheral target size in each trial.

In the case of saccadic adaptation, we identified in each trial the saccade that triggered the display change (step or peripheral stimulus disappearance in training and test trials, respectively). Saccades were detected using the Eyelink II (SR Research, Mississauga, Canada), with velocity threshold set at 30°/s and acceleration threshold set at 8000°/s^2^. The amplitude of the saccade in the direction of the target was then converted to gain by dividing it by the presaccadic target eccentricity (16°). We discarded from the analysis the trials in which the gain of the saccade was less than 0.5 (2.2% of total) or more than 1.5 (0.2% of total).

### Results

**Figure 6.**
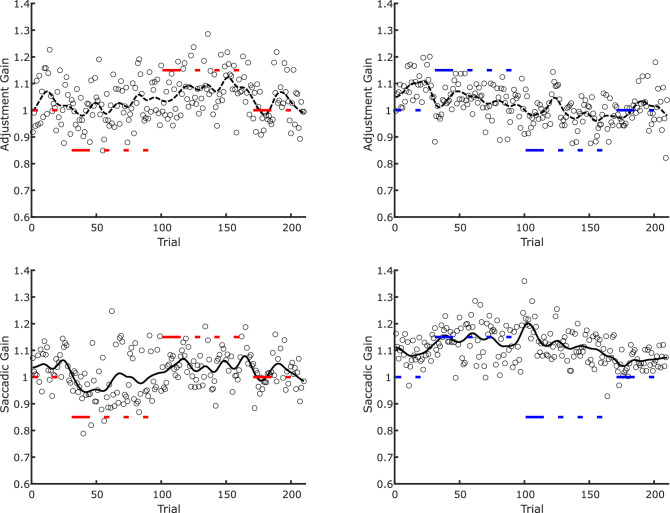
Examples of raw data in Experiment 2. Top panels: adjustment gain in trans-saccadic recalibration trials. Bottom panels: saccadic gain in the saccade adaptation trials. Left panels: decrease-increase and inward-outward sessions. Right panels: increase-decrease and outward-inward sessions. Black dots represent individual adjustments/saccade amplitudes in one given trial. The black lines represent the smoothed (Gaussian kernel with *SD* = 4 trials) time course. The thick red and blue horizontal lines represent the gain of the trans-saccadic change (size or position) in the respective trials. The interruptions in the red and blue lines denote test trials in which either no saccade was executed, or the stimulus disappeared during the saccade.

**Figure 7.**
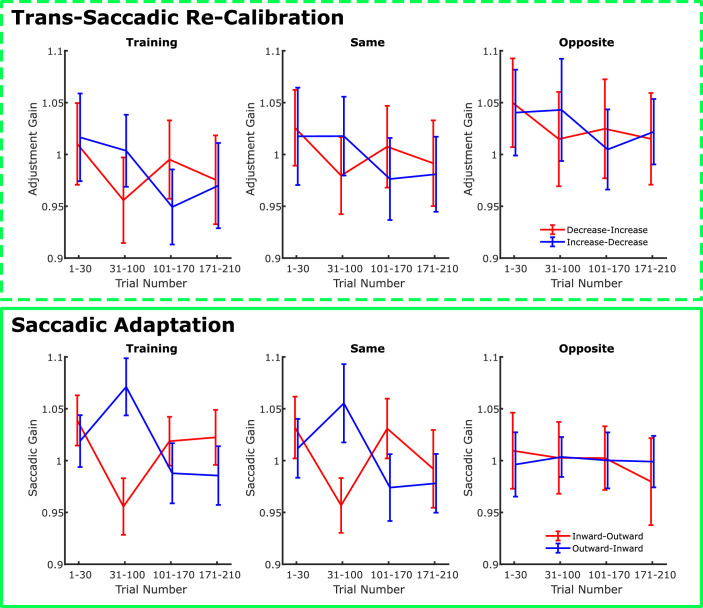
Overall results in Experiment 2 for the trans-saccadic recalibration and saccadic adaptation trials. Data are reported for training trials, for test trials at the same location of training, and for training trials at the opposite location in different panels. Furthermore, the results are split based on the sequence of training trials in a given session. Notice that all plots show a clear crossover interaction between trials 31-100 and 101-170, except for the opposite location in the saccadic adaptation session (bottom right). This crossover interaction is indicative of the effect of training. The fact that it is absent at the opposite location in the saccade adaptation session indicates that saccadic adaptation does not transfer spatially, whereas trans-saccadic recalibration of perceived size does. Error bars are between-observer *SEM*.

Individual examples of the time course of trans-saccadic recalibration and saccade adaptation from Experiment 2 are depicted in Figure 6. To compute an aggregate measure of trans-saccadic learning, we averaged the gain values across the four segments of each session (trials 1 to 30 without trans-saccadic size change or target step, trials 31 to 100, and trials 101 to 170 with opposite trans-saccadic manipulations, and trials 170 to 210 again without trans-saccadic manipulation). In Figure 7 we plotted the averaged data for training trials, test trials at the same location, and test trials at the opposite location, respectively, and separately for the different training sequences (decrease-increase vs. increase-decrease for trans-saccadic recalibration and inward-outward vs. outward-inward for saccadic adaptation).

A quick inspection of the plots in Figure 7 reveals a clear crossover interaction in the gain values in the middle of the session in all plots but the one relative to saccade adaptation at the opposite location. This crossover interaction is the diagnostic sign of learning in our paradigm, as it mirrors the reverse order of the training manipulation. The fact that it is absent specifically in the case of saccadic adaptation at the opposite locations seems to suggest that saccadic adaptation acquired at one location, contrary to trans-saccadic recalibration of perceived size, does not transfer to a mirror location in the other hemifield.

Notice also that there is a general tendency to overshoot in the first trial block of the saccade adaptation session. This might have to do with the fact that observers were trained to produce one clear saccadic movement toward the target in the previous session, to ensure that the trans-saccadic manipulation could be applied as the saccade was in flight. Furthermore, notice that the relative amplitude of saccades at the end of the session is generally the same in trials 101-170 as it was in trials 1-30, or slightly lower. This is compatible with the idea that saccade amplitudes are determined by the adaptation effect and by a general tendency to saccade amplitude decrease (Cassanello et al., 2019). This decrease is probably less evident in our paradigm owing to the fact that observers were already producing saccades in the previous trans-saccadic recalibration session.

**Figure 8.**
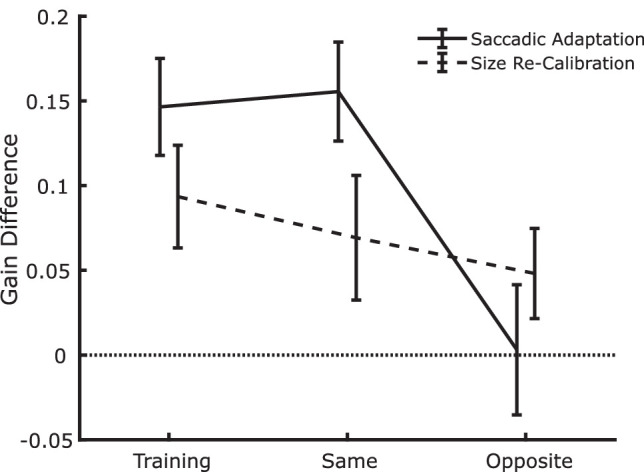
Summary plot indicating the strength of size recalibration and saccadic adaptation computed as the difference in gain between the trials in which the trans-saccadic change was increase/outward and the trials in which it was decrease/inward. Data are plotted as a function of the type of trial (training, test at the same location, test at the opposite location). The results clearly show an interaction in which the gain difference is positive (as expected) in each case, except at the opposite location for saccadic adaptation. Error bars are between-observer *SEM*.

To summarize the main finding of the experiment, and to allow for a direct statistical analysis of the main experimental question, we further aggregated the data by computing, for each panel in Figure 7, the difference in the average gain in the segments in which the training trans-saccadic change was increase/outward, and the average gain when the training was decrease/inward. The values are shown in Figure 8. Evidently, all values are positive (as expected if the training affects the adjustments/saccadic amplitudes), and mostly larger in the case of saccadic adaptation, except for the opposite position, in which there is no sign of saccadic adaptation but there is still size recalibration. For comparison, if learning would be complete, the total gain difference would be 0.3 (twice the 0.15 trans-saccadic manipulation), so when present, saccadic adaptation reaches 50% of the maximum expected effect and size recalibration approximately 25%. The general impression was confirmed by an analysis of variance with task (size recalibration vs. saccade adaptation) and trial type (training, test at the same location, and test at the opposite location) as factors, which revealed a significant two-way interaction, *F*(2,22) = 11.867, *p* < 0.001, *ηp*² = 0.519. This was further explored by performing one-sample *t*-tests against a value of 0 in each cell of the experimental design (Table 1), which evidenced significant effects of learning in all but the case of saccadic adaptation at the opposite location, again confirming that spatial transfer of trans-saccadic learning at mirror locations takes place in the case of size recalibration but not in the case of saccadic adaptation.

**Table 1.** Individual *t*-test results. All cells show a significant effect of training except for the one corresponding to the test trials at the opposite location in the case of saccadic adaptation.

Task	Trial type	*t (11 d* *f* *)*	*p* (Bonferroni corrected)
Saccadic adaptation	Training	10.22	<0.001
	Same	10.64	<0.001
	Opposite	.16	=1
Size recalibration	Training	6.17	<0.001
	Same	3.76	=0.018
	Opposite	3.61	=0.024

## Discussion

In two experiments, we investigated the dynamics and spatial specificity of trans-saccadic recalibration of perceived size. In Experiment 1, we found that sinusoidal oscillation of trans-saccadic change produces sinusoidal oscillations in the gain of size adjustments with a lag of a few trials. In Experiment 2, we found that trans-saccadic learning of perceived size acquired at one location transfers to the opposite hemifield, whereas no transfer is observed for saccadic adaptation. Overall the results clarify that these two forms of learning, albeit taking place under similar experimental conditions and within the same temporal range, are qualitatively different.

Experiment 1 (Time) was inspired by the paradigm used by Cassanello, Ohl, and Rolfs (2016) in their investigation of the temporal dynamics of saccadic adaptation. Apart from the obvious aspect that Cassanello et al. (2016) used trans-saccadic target steps to modify the amplitude of saccades, whereas we used trans-saccadic size changes to modify reported peripheral size, the two paradigms differed in a few more specific points. First, both studies used eye movements of a fix amplitude, but the current paradigm required observers to consistently produce eye movements with a fixed starting location (center of the screen) and a fixed landing location (left or right). The Cassanello et al. paradigm instead required observers to either make horizontal saccades, toward the left or the right (“two-way adaptation”), or to execute saccades toward any possible direction including oblique ones (“global adaptation”). Second, Cassanello et al. (2016) were able to test multiple frequencies for the trans-saccadic step modulation. In our one-stimulus-stream experiment, a full cycle of the sinusoidal modulation encompassed 100 trials, which is equivalent to the six cycles per block condition in their previous study. Note, however, that because participants must adjust the size of the stimuli, the trials last much longer (around five trials per minute) than trials in saccade adaptation (up to 100 trials per minute), meaning that if the frequency was defined in terms of cycles per minute, all frequencies in the saccade adaptation study would be much higher than in the current study. With these caveats in mind, the condition in the study by Cassanello et al. (2016) that is most comparable to our study (i.e., two-way saccadic adaptation with 100 trials per cycle) produced oscillations in the saccadic gain that had average amplitude of 12% relative to the amplitude of the modulation of the trans-saccadic step. The average lag was 10.49 trials. These values are of the same order of magnitude as the values that we observed in the current study (20.9% and 5.58 trials, respectively). If anything, a comparison between the two studies suggests that trans-saccadic recalibration of perceived size might be a slightly faster process than saccadic adaptation.

In our Experiment 2 (Space), we used a similar paradigm to test another possible difference between saccadic adaptation and perceptual recalibration: the degree of spatial specificity. Valsecchi and Gegenfurtner (2016) found that size recalibration is a global phenomenon. In contrast, Herwig and colleagues (2018) found spatially specific trans-saccadic learning, induced by systematic changes in spatial frequency. The results of Valsecchi and Gegenfurtner (2016) seem at odds with what is commonly found for saccadic adaptation, which does not generalize to very different saccade vectors (Alahyane, Fonteille, et al., 2008; Frens & Van Opstal, 1994; Hopp & Fuchs, 2004; Noto et al., 1999). In the current study, therefore we decided to test spatial generalization for size recalibration and saccade adaptation using a comparable paradigm and with the same observers. The results unequivocally confirmed the previous findings of spatial transfer for size recalibration (Valsecchi & Gegenfurtner, 2016) and no spatial transfer for saccadic adaptation (Alahyane, Devauchelle, et al., 2008; Frens & Van Opstal, 1994; Hopp & Fuchs, 2004; Noto et al., 1999). Rather than being confined to a recalibration field, recalibration seems to spread at least to the mirror location in the opposite hemifield^1^. There might be functional reasons for this. Saccadic adaptation can deal with problems, such as general fatigue, which will produce a general reduction in saccadic amplitudes, but also with the weakening of a particular extraocular muscle, which will produce direction-specific changes in saccadic amplitude (Snow, Hore, & Vilis, 1985). On the other side, the central-peripheral anisotropies in the visual field, which we think make trans-saccadic recalibration necessary, are prominently eccentricity-dependent but hemifield-independent.

It remains an open question why Herwig and colleagues (2018) found spatially selective trans-saccadic learning for perceived spatial frequency. One possibility is that oblique saccades or spatial frequency (as used in their study) have a special status. Another possibly relevant aspect is that observers congruently trained two opposite locations at the same time, whereas here and in Valsecchi and Gegenfurtner (2016), observers trained only one location. It seems odd, however, that being exposed to less localized training would produce a more spatially selective form of learning. For sure Rolfs and colleagues (2010) showed that the opposite is the case for saccadic adaptation. At present we can speculate that the most relevant difference between the two paradigms is the focus on object-specific learning in Herwig and colleagues (2018). Although parametrically changing the size of circular objects at one location across saccades might produce global recalibration, selectively associating one specific object to a specific trans-saccadic spatial frequency change might induce a spatially selective form of learning.

Additionally, this study leaves open what role time plays in the acquisition of size recalibration. As we mentioned before, the duration of each trial was much longer in the present study than in a typical motor learning task, in which subsequent movements can be executed with almost no interruption, and significantly longer than in the study by Valsecchi and Gegenfurtner (2016). One possible way of investigating the temporal integration of trans-saccadic feedback would be to test perception only after a number of trials in which only the saccadic task is executed. Those saccade-only trials could be distributed over a varying time interval, thus manipulating the saccade and feedback rate.

On a more general level, we think our findings reflect the large degree of complexity of what happens when our trans-saccadic visual predictions are violated. It is quite clear that even saccadic adaptation per se might be in fact due to multiple functionally distinct and possibly unrelated processes. For instance, although both reactive and voluntary saccades can be adapted, adaptation does not transfer between the two (e.g., Deubel, 1995; Erkelens & Hulleman, 1993) and distinct areas of the cerebellum are responsible for the adaptation of reactive and voluntary saccades (Alahyane, Fonteille, et al., 2008). Furthermore, although adaptation of reactive saccades only affects the perceived position of flashed stimuli, adaptation of voluntary saccades induces mislocalization of stationary stimuli as well (Zimmermann & Lappe, 2009). A further relevant distinction is between inward and outward adaptation. Outward adaptation is both slower and less complete compared with inward adaptation (e.g., Miller, Anstis, & Templeton, 1981; Semmlow, Gauthier, & Vercher, 1989). Selective impairment of inward adaptation can be observed after thalamic lesions (Zimmermann, Ostendorf, Ploner, & Lappe, 2015) and inward adaptation seems to involve changes in the speed profile of saccades, whereas outward-adapted saccades have identical speed profiles to regular saccades of the same amplitude (Catz, Dicke, & Thier, 2008; Ethier, Zee, & Shadmehr, 2008). More relevant to our study, only outward adaptation is associated with a change in spatial localization during fixation (Hernandez, Levitan, Banks, & Schor, 2008; Zimmermann & Lappe, 2010), but both inward and outward adaptation produce changes in the distance to which a stimulus is perceived to extend from the fixation point (Garaas & Pomplun, 2011). Zimmermann and Lappe (2016) developed a model based on the assumption that saccadic adaptation can take place both through changes in the circuit connecting the superior colliculus, cerebellum and brainstem saccade generator, and through changes in the cortical motor command (in the frontal eye fields). Only the changes taking place at the cortical level would be associated with changes in the perceptual representation of visual space, which would explain why saccadic adaptation alters perception of space mostly in the case of voluntary saccades.

We can speculate that trans-saccadic recalibration of perceived size only shares some processes with saccadic adaptation, specifically those related to trans-saccadic prediction and prediction error evaluation. These shared processes might explain why the extent of saccadic adaptation and recalibration correlate, albeit rather weakly, across observers (Bosco et al., 2015), while at the same time having qualitatively different patterns of spatial generalization, which might reflect the qualitatively different processes that might subtend the plastic changes that compensate for space and object distortions. In particular, it seems highly unlikely that trans-saccadic recalibration, which we induced always by means of voluntary saccades, would rely on subcortical structures, such as the superior colliculus-cerebellum circuitry responsible for the adaptation of reactive saccades (Zimmermann & Lappe, 2016). It is an open question whether recalibration of the perceived size of flashed stimuli, induced within the context of reactive saccades, takes place, and if it does, whether it transfers to static stimuli.

As we discussed earlier, one of the findings that motivated us to investigate the relationship between trans-saccadic size recalibration and saccade adaptation is the finding by Bosco and colleagues (2015) that saccade amplitude, as well as perceived size, can change when the center of mass of the stimulus changes trans-saccadically. In a similar vein, Lavergne and colleagues (2010; 2011) showed that saccades executed within an object can be adapted by a trans-saccadic change in object size, so that saccades become longer if the object becomes larger and vice-versa. The type of trans-saccadic learning that we observe in our size-recalibration paradigm is in a way further from saccade adaptation because the physical manipulation that we apply trans-saccadically has no effect on saccade amplitudes in the first place. Saccades are larger if they are executed to a stimulus whose (peripheral) center of mass is shifted outward, and they are larger if they take place within a larger object. In our case, saccades directed toward a peripheral stimulus are unrelated to the size of the stimulus itself^2^. It is still an open question whether a trans-saccadic change in size that also involves a trans-saccadic change in the stimulus center of mass can produce a trans-saccadic recalibration of perceived size with a pattern of spatial specificity closer to that of saccade adaptation, or whether the two would be dissociable within the same paradigm.

One final question that we did not address in our paradigm is whether saccade adaptation produces a change in the perceived size of objects. Garaas and Pomplun (2011) showed that saccade adaptation produces a change in the perceived size of a stimulus that protrudes from the fixation point in the adapted direction. Our stimuli differ from theirs in that they are entirely located in the periphery, so that size cannot be coded as distance from fixation. Finding a change in their perceived size after saccade adaptation could provide strong support to the idea that saccade adaptation can warp perceptual space beyond the perception of distance and position. If this were found to be the case exclusively for outward adaptation, that would strengthen the idea that this type of adaptation is more directly connected to higher-level mechanisms. Unfortunately, we did not ask our observers to report perceived size in our saccade-adaptation trials. This can be addressed in future experiments.

## Conclusions

Our findings reveal that trans-saccadic recalibration of perceived size occurs rapidly in response to an oscillating trans-saccadic size change. They confirm that, contrary to saccade adaptation, size recalibration extends to mirror locations. Generally speaking this points to both similarities, in terms of temporal dynamics, and differences, in terms of spatial selectivity, between this form of perceptual trans-saccadic learning and visuomotor trans-saccadic learning revealed by saccadic adaptation. Although these phenomena can certainly co-occur when the trans-saccadic change used in a given paradigm requires both perception and saccade programming to adapt (Bosco et al., 2015), they remain functionally distinct.

## Footnotes

1 Additional testing conducted using the Valsecchi and Gegenfurtner (2016) paradigm indicate that on the horizontal axis trans-saccadic recalibration is generalized to larger eccentricities, and to a lesser extent to smaller eccentricities independently from the hemifield (unpublished data).

2 For instance in the training trials of the size recalibration sessions in Experiment 2, stimulus size had no effect on saccade amplitude: *F*(4,44)=.655, *p*=.626.
